# Oxidative Stress in Neurodegenerative Diseases: From Preclinical Studies to Clinical Applications

**DOI:** 10.3390/jcm9041223

**Published:** 2020-04-24

**Authors:** Andrea Tarozzi

**Affiliations:** Department for Life Quality Studies, University of Bologna, 47921 Rimini, Italy; andrea.tarozzi@unibo.it

Oxidative stress plays an important role in the pathogenesis of several different neurodegenerative diseases (NDDs), such as Alzheimer’s disease (AD), Parkinson’s disease (PD), Huntington’s disease, amyotrophic lateral sclerosis (ALS) and multiple sclerosis (MS) [1,2]. In particular, recent studies indicate that various forms of crosstalk between oxidative stress, inflammation, proteostasis impairment, mitochondrial dysfunction, and other neurodegenerative events, occur in onset or during the progression of neurodegenerative diseases [3,4]. In this regard, findings in cell, organism, and animal models, as well as biomarker studies in humans have also suggested a relationship between redox status regulation in neurodegenerative processes and neuroprotection. As a result, neuroprotective molecules with antioxidant properties have received increasing attention as therapeutic and preventive interventions for NDDs [5,6]. The manuscripts reported in this Special Issue deal with different aspects including new interplays between oxidative stress and critical molecular and cellular pathways in NDDs, potential redox biomarkers of disease progression and neuroprotective effects of endogenous, natural and synthetic molecules against the oxidative stress and inflammation in experimental models of neurodegeneration.

Lin et al. [7] performed a combined experimental approach of next-generation sequencing with bioinformatics analysis and experimental studies on human astrocytes treated with indoxyl sulfate (IS), a uremic toxin that increases the risk of cognitive impairment in chronic kidney disease (CKD). In this regard, IS triggered astrocyte apoptosis through oxidative stress induction, nuclear translocation of nuclear factor (erythroid-derived 2)-like 2 (Nrf-2), extracellular-signal-regulated kinase (ERK), c-Jun N-terminal kinase, and p38 inhibition in astrocytes. In particular, the decreased ERK kinase phosphorylation was mediated by the upregulated dual-specificity phosphatase 1, 5, 8, and 16. They concluded by suggesting that decreasing the IS levels could prevent the neurological complications recorded in CKD. In their manuscript, Bordoni et al. [8] demonstrated an increase of DNA damage evoked by oxidative stress in peripheral blood mononuclear cells of subjects with sporadic ALS (sALS), and SH-SY5Y model of ALS disease. In this regard, they focused on the activation of ataxia-telangiectasia-mutated (ATM)/Chk2 and ATM- and Rad3-related (ATR)/Chk1 DNA damage response pathways, which lead to phosphorylation of superoxide dismutase 1 (SOD1). They found that the phosphorylation of SOD1 by Chk2 activates its translocation in the cell nucleus, where SOD1 protects DNA from oxidative damage. They therefore supposed that this physiologic pathway is altered in subjects with sALS and suggested that it could represent an innovative therapeutic target for sALS.

Other manuscripts reviewed the emerging role of oxidative stress-responsive proteins, such as sestrins, in various neurological diseases, protein DJ-1 in PD and mitofusin-2 (Mfn2) in AD. Chen et al. [9] reported that several studies in NDDs focused on sestrin2, but information regarding the roles of other sestrins, including sestrin1 and sestrin3, in the nervous system is still limited. In particular, they highlighted that sestrin2 can indirectly counteract the oxidative stress by regulating mTOR to enhance autophagy, or more specifically mitophagy. The multiple biological function of sestrin2, in terms of indirect antioxidant activity and autophagy promotion to remove the aggregated proteins, suggests new opportunities for the treatment of NDDs caused by misfolded protein toxicity, such as AD, PD, HD and ALS. In their review, Repici et al. [10] provided an overview of the DJ-1 relevance as a potential biomarker for PD, as well as a varied range of NDDs. Due to its active role in neuroprotection from oxidative stress, they suggested that DJ-1 also represents an ideal target for therapeutic intervention, but further studies are necessary to clarify whether DJ-1 targeting compounds can either stabilize the active DJ-1 form or increase DJ-1 activity to obtain neuroprotection. Sita et al. [11] reviewed the mitofusins that regulate the mitochondrial fission/fusion dynamics in AD. Among these proteins, they focused on the role of mitofusin 2 (Mfn2) in mitochondrial dynamics and in the crosstalk between mitochondria and the endoplasmic reticulum. In this context, recent studies highlighted a decrease of Mfn2 expression in various AD models suggesting an imbalance of mitochondrial dynamics as well as bioenergetic metabolism, which can contribute to AD pathogenesis. However, further studies are needed to define the Mfn2 druggability for the development of neuroprotective therapies in AD.

Among neurodegenerative events, chronic inflammation can exacerbate the oxidative stress in the cells, leading to oxidation and damage of cellular components, increased inflammation and activation of neuronal death pathways [4]. In this regard, García-Revilla et al. [12] reviewed the microglia polarization states under conditions of neurodegeneration and their leading role in oxidative stress. They considered the classic microglia polarization states (M1, pro-inflammatory; M2, immunosuppressive) of microglia and a new microglia subpopulation named disease-associated microglia (DAM). Two microglia receptors, toll-like receptors (TLRs) and triggering receptors expressed on myeloid cells-2, TREM2, are believed to generate either a pro-inflammatory (highly pro-oxidant) or an anti-inflammatory DAM phenotype, respectively. However, the recent identification of endogenous multiple ligands of these receptors suggests the existence of complex microglia responses and the possibility that different microglia subtypes may coexist. In particular, different DAM subtypes do not exclude the existence of pro-oxidant DAM.

As previously highlighted, the identification and use of oxidative stress biomarkers in NDDs could be useful for the development of new drugs as well as for the early diagnosis and demonstration of drug efficacy in clinical studies. Padureanu et al. [13] evaluated the oxidative stress/inflammatory level in peripheral blood of subjects with relapsing-remitting MS using conventional plasma markers, including thiobarbituric acid reactive substances, protein carbonyl level, total antioxidant capacity and neutrophil/lymphocyte ratio. Among these plasma markers, neutrophil/lymphocyte ratio was associated with a decreased antioxidant capacity, even in the early stage of neuronal damage, suggesting its use as a biomarker of pro-inflammatory status of the relapse-remitting stage of MS. Klimiuk et al. [14] considered oxidative stress biomarkers in saliva and blood of subjects with different degrees of dementia progression. In particular, they recorded that the depletion of antioxidant defenses as well as the levels of oxidative and glycoxidative damage increased in both saliva and blood during the progression of dementia and decline of cognitive functions. Notably, the salivary reduced glutathione (GSH) correlated not only with the severity of dementia, but also with GSH concentration in blood. They therefore proposed GSH as a potential salivary non-invasive biomarker of cognitive impairment. On the subject of non-invasive biomarkers, Maciejczyk et al. [15] reviewed the latest knowledge concerning the salivary redox biomarkers used in the diagnosis of NDDs. In this regard, the concentrations of many redox biomarkers in saliva correlated with their content in blood as well as the degree of disease progression, which makes them non-invasive indicators of NDDs. Salivary advanced glycation end products, AGE, and GSH are particularly noteworthy in the diagnosis of NDDs, and together with the salivary heme oxygenase, HO-1, appear to be especially interesting for the diagnosis of the early stages of PD.

Among the research manuscripts of this Special Issue, some papers evaluated the antioxidant and anti-inflammatory effects of different molecules in several in vivo models of neurodegeneration. Hong et al. [16] evaluated the effects of environmental lighting conditions regulating endogenous melatonin production on neural repair, after spinal cord injury (SCI) in rats. They recorded that a high level of melatonin, under constant dark condition, plays an anti-inflammatory and antioxidative role in the acute phase of SCI, leading to early oligodendrogenesis, excitatory synaptic formation, and axonal outgrowth mediated through NAS-TrkB-AKT/ERK signal transduction. They suggested that endogenous melatonin, under light/dark control, is a crucial factor that affects neural repair after SCI. Khan et al. [17] showed the neuroprotective effects of caffeine against the neurodegenerative effects elicited by cadmium in mice. In particular, the treatment with caffeine attenuated the neuronal loss, neuroinflammation, and cognitive deficits induced by cadmium. In addition, they demonstrated that caffeine exerts neuroprotection via regulation of Nrf-2- and nuclear factor-kB-dependent mechanisms in the hippocampal HT-22 and microglia BV-2 cell lines, respectively. Beggiato et al. [18] evaluated the antioxidant and neuroprotective effects of chronic treatment with an ultramicronized formulation of N-palmitoylethanolamide (um-PEA) in an animal model of Alzheimer’s disease (3xTg-AD mice). The results indicated that um-PEA by oral administration reaches the mice brain. Further, the treatment with um-PEA reduced the neuroinflammation and oxidative stress, and rescued the cognitive deficit in 3xTg-AD mice. They remarked that the use of PEA already authorized for human consumption supports its rapid translation in clinical practice. Lastly, Ahmad et al. [19] assessed the anti-inflammatory and neuroprotective effects of flavonoid fisetin in a murine model of neuroinflammation induced by lipopolysaccharide (LPS). The fisetin abolished the oxidative stress and gliosis, neuronal death and synaptic/memory deficits evoked by LPS. In particular, fisetin inhibited several markers of neuroinflammation such as the activation of TLR4/CD14 signaling pathway and pro-inflammatory cytokines (i.e., tumor necrosis factor-α, interleukin-1β and cyclooxygenase 2). They suggested the potential use of fisetin for the prevention and treatment of neurodegenerative diseases.

In conclusion, the manuscripts published in this Special Issue highlight recent advances in knowledge of the oxidative stress’s contribution to various NDDs as well as novel antioxidant strategies of neuroprotection for NDDs.
